# The Impact of Depression on Quality of Life in Caregivers of Cancer Patients: A Moderated Mediation Model of Spousal Relationship and Caring Burden

**DOI:** 10.3390/curroncol29110639

**Published:** 2022-10-27

**Authors:** Yoonjoo Kim

**Affiliations:** Department of Nursing, College of Healthcare Sciences, Far East University, Eumseong-gun 27601, Chungcheongbuk-do, Korea; yjbaraem@gmail.com; Tel.: +82-43-880-3242

**Keywords:** care burden, cancer, caregiver, depression, moderated mediation, spouse, quality of life

## Abstract

Family caregivers play an important role in managing and supporting cancer patients. Although depression in family caregivers is known to negatively affect caregiver health, the mechanism by which it affects caregivers is not clear. The purpose of this study was to explore the influence of depression on quality of life (QoL) in family caregivers of patients with cancer. Specifically, this study examined (1) whether caring burden mediates the relationship between depression and QoL, and (2) how this mediating effect varies depending on the caregiver’s relationship with the patient. This study performed a secondary analysis on cross-sectional survey data. Ninety-three family caregivers of cancer patients were included in the study. Moderated mediation analyses were conducted using PROCESS macro with the regression bootstrapping method. The moderated mediation models and the indirect effect of caregiver depression on QoL through caring burden were significantly different depending on caregivers’ relationships with patients (i.e., spousal or non-spousal). Specifically, the indirect effect of caregiver depression on QoL was greater for the patient’s spouse than for other family caregivers. Healthcare providers should focus on identifying caregivers’ depression and relationship with the patient and offer tailored support and intervention to mitigate the caring burden and improve the caregivers’ QoL.

## 1. Introduction

Over the years, cancer has emerged as one of the major causes of mortality worldwide. The incidence and survival rates of cancer have increased over time [1,2]. In recent years, the medical environment has shortened hospital stays; as outpatient or home treatment rises, families play an increasingly important role as caregivers in managing and supporting cancer patients [3,4]. Cancer is effectively a family problem because it forces continuous changes in the lives of patients and their families [5]. Family caregivers are primarily responsible for caring for cancer patients and providing physical, mental, social, and economic support, as well as performing therapeutic roles, such as symptom management and monitoring, without any remuneration [5]. Although family support contributes to improving outcomes in cancer patients, family caregivers encounter multifaceted burdens in their role and lifestyle including employment changes, disrupted schedules, and challenges to their emotional well-being [6]. Owing to increased responsibilities, they cannot afford to take care of themselves and tend to hide and suppress negative emotions [4,6]. As a result, family caregivers of cancer patients experience increased psychological distress [4,7] and poor physical health [8,9].

Research has shown that family caregivers of cancer patients commonly experience depression [4,10]. According to a meta-analysis of 30 studies, the prevalence of depression among those providing care to cancer patients was 42% [10], and caregiver depression was sometimes more severe than that in patients [11]. Depression in family caregivers leads to an increased burden related to care needs [12], poor physical health [9], impaired ability to provide care [13], and poor quality of life (QoL) [10,14]. Caregiver depression is known to have a negative effect on the health of both patients and caregivers [15]. However, thus far, the literature has focused on the prevalence of depression in family caregivers and care-related factors affecting depression [10,16]. Prior research investigating the impact of depression on QoL in caregivers of cancer patients has rarely considered the interplay of multiple caregiving contexts concurrently, especially the effects of caring burden and family relationship with the patient. This study focuses on caregiver depression and whether its indirect effect on caregivers’ QoL is mediated by their caring burden and the moderation effect of their relationship with the patient.

### 1.1. Depression and QoL in Family Caregivers of Cancer Patients

The relationship between depression and QoL in family caregivers of patients with cancer is well established [10,14]. A systematic literature review and meta-analysis of 30 studies on caregivers of cancer patients investigated the factors affecting depression and QoL [10]. A cancer diagnosis in the family itself is a major cause of caregiver depression, and it has been shown that caregivers’ age, sex, employment status, education level, relationship with the patient, and caring burden are all associated with poor QoL. In addition, a literature review of 26 studies on the caring experience of Korean family caregivers showed that the greater the depression or the caring burden, the lower the QoL [4].

QoL is a multidimensional factor, encompassing physical, emotional, and social well-being. Unmet needs, lack of social support, high symptom burden for cancer patients, lack of care for caregivers during treatment, and caregivers’ psychological and physical symptoms can lead to reduced QoL among family caregivers [7,10,12]; however, support and interventions for family caregivers improve QoL [3,7]. It is necessary to pay attention to caregivers’ issues, as a decline in family caregivers’ QoL can negatively affect the quality of care they can provide [15].

The caring burden is also a multidimensional factor, reflecting the subjective and objective levels of the burden of caring for patients, as perceived by caregivers [17]. Caregivers experience degradation of physical health, including sleep disturbance and fatigue, due to their caring burden [8,13]; alarmingly, the higher the caring burden, the higher the risk of death for caregivers [18]. In addition, long-term care provision increases role overload and caring burden, resulting in a lower level of QoL among caregivers compared with the general population [4,7,12,19]. Thus, the caring burden is an important determinant of caregivers’ QoL [4,7,10]. While the relationship between depression, caring burden, and QoL in family caregivers of cancer patients is known, the direction of this relationship is not clear. Understanding the relationship between family caregivers’ QoL and the factors influencing it can help adjust the caring burden and improve QoL in caregivers.

Family caregivers include the patient’s spouse, adult child, parents, siblings, or relatives [5]. The relationship between the patient and caregiver has been shown to influence QoL in family caregivers [10]; family caregivers caring for newly diagnosed lung cancer patients were more likely to be depressed if they were a spouse than if they had a different relationship with the patient [7]. In a study of 897 Korean family caregivers [20] and family caregivers of terminally ill cancer patients [19], spouses had higher levels of depression and caring burden than other family caregivers. As such, in terms of relationship with the patient, depression and caring burden were higher and QoL was lower among caregivers who were the patients’ spouses.

A recent systematic literature review of physical and psychological morbidity in cancer patients and caregivers confirmed that their health is interdependent [15]. As many factors, including the health status of family caregivers in cancer patient management, affect patient health outcomes, it is necessary to understand the factors and mechanisms affecting caregivers’ QoL.

### 1.2. Theoretical Framework

**Figure 1:**
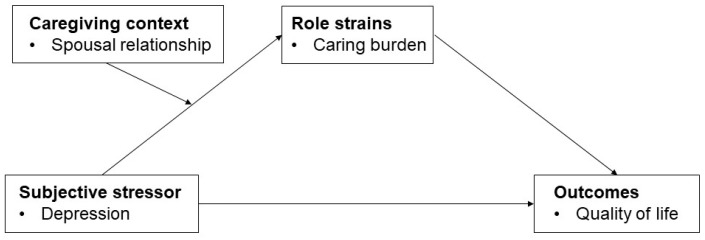
The conceptual model of the study.

The conceptual model of this study (Figure 1) was developed based on the stress process model [21], which states that stressors faced by family caregivers negatively affect their outcomes. It is assumed that primary stressors, both objective and subjective, influence secondary role strains, which in turn may directly or indirectly affect outcomes (e.g., well-being). The model also notes that the caregiving context may further either reinforce or buffer the effects of primary stressors on secondary role strains. In this study, the model was developed with depression as a subjective stressor, caring burden as a secondary role strain, and QoL as the outcome shaped by the stressor and role strain. Furthermore, the caregivers’ relationship with the patient was assumed to reinforce or buffer the effects of caregiver depression on care burden and QoL.

This study aimed to examine whether the relationship between patients and caregivers moderates the mediating effect of the caring burden on the relationship between depression and QoL in caregivers. This was conducted in two steps, examining (1) whether depression affects QoL through caring burden (i.e., a mediation model) and (2) whether the strength of the mediated effect through caring burden varies according to the relationship with the patient (i.e., a moderated mediation model). Specifically, we hypothesized that depression is associated with poor QoL (i.e., a direct effect) and that this relationship is mediated by the effect of the caring burden on QoL (i.e., an indirect effect through caring burden). Our theoretical framework asserts that the caregivers’ relationship with the patients also influences the mediation model. In particular, we hypothesized a stronger direct and indirect relationship between depression and poor QoL when the caregiver was a spouse compared with caregivers who were not spouses (i.e., a moderated mediation model).

## 2. Materials and Methods

This was a secondary analysis of cross-sectional survey data from 93 family caregivers of cancer patients from outpatient clinics or inpatient wards of university hospitals in Korea. To date, two studies have been published using these data; these studies examined (a) the association between inflammatory cytokines and caregiving distress in family caregivers [8] and (b) the mediating effect of QoL on the relationship between perceived stress and immune function [22].

### 2.1. Sample and Data

The sample included 93 family caregivers of patients undergoing treatment for cancer. Family caregivers were involved in multiple aspects of cancer treatment as the family members were most responsible for caring for cancer patients; they spent more time with the patient than other family members and were adults aged 20 years or above. Caregivers with uncontrolled psychiatric disorders or autoimmune diseases were excluded. Participants were recruited from March to December 2017, through recruitment notices in the hospital. Participation in the survey was voluntary. Ethics approval for this study was obtained from the institutional review board of the affiliated university (approval number: 2017-1).

The final sample size for this study was determined using the G*Power 3.1 program [23]. Based on the multiple regression analysis, the significance level (α) was 0.05, effect size (*f*^2^) 0.15, and power (1-β) 0.89, when calculated as five independent variables.

### 2.2. Measures

#### 2.2.1. Depression

Depression was assessed using the Center for Epidemiological Studies Depression Scale (CES-D) [24], which indicates the level of depression experienced in the past week. It comprises 20 items, and each item is evaluated on a four-point Likert scale from 0 (almost none) to 3 (always). The total score ranges from 0 to 60, with higher scores indicating higher levels of depression. A score of 16 or higher indicates clinically significant depression [24]. In this study, Cronbach’s α was 0.89.

#### 2.2.2. Caring Burden

The caring burden was assessed using the Korean version of the Zarit Caregiver Burden Interview (K-ZBI) [17]. This scale comprises 22 items rated on a five-point Likert scale ranging from 0 (not at all) to 4 (always). It assesses the following five domains: burden on relationships, emotional well-being, social and family life, finances, and loss of control over one’s life. The total score ranges from 0 to 88, with higher scores indicating a higher caring burden. In this study, Cronbach’s α was 0.93.

#### 2.2.3. Quality of Life

QoL was assessed using the Korean version of the Caregiver Quality of Life Index-Cancer (CQOLC-K) [25,26]. This scale comprises 35 items rated on a five-point Likert scale from 0 (not at all) to 4 (very much). The total score ranges from 0 to 140, with higher scores representing better QoL. In this study, Cronbach’s α was 0.89.

#### 2.2.4. Family Relationship with Patient

In this study, participants were classified into two groups: those in a spousal relationship with the patient or a non-spousal relationship, including the patient’s parents, adult child, and siblings [7,19,20].

### 2.3. Data Analysis

Data were analyzed using SPSS version 26.0 (IBM Corp., Armonk, NY, USA), and a *p*-value of 0.05 was set as the significance level for all statistical tests. Descriptive statistics (mean, standard deviation, frequency, and percentage) were used to describe the general characteristics and measured variables for all participants. Comparisons across groups were performed using *t*-tests. The relationships between the research variables were investigated using Pearson’s correlation and point-biserial correlation.

To address the specific aim, the model-building procedure proceeded from a simple model to a more complex one, using PROCESS macro 3.5.3. [27]. A mediating variable can be conceptualized as a third variable that intervenes in the relationship between two or more variables and acts as the mechanism by which one variable influences another. However, the process of mediation may vary depending on different situations (i.e., moderating variables). In other words, if the effect of *X* on *Y* via *M* depends on the moderating variable, it can explain the mechanism that connects *X* and *Y*. This combined association is called a moderated mediation model (conditional process model) [23]. The mediating model used Model 4 [23] to confirm whether the influence of depression (*X*) on QoL (*Y*) was due, in part, to the presence of a caring burden (*M*). Once the simple mediation model was confirmed, a moderated mediation model was run using Model 7 [23]. This model tested whether there were direct or indirect relationships between depression (*X*) and QoL (*Y*) that were influenced by caregivers’ relationship with the patients (*W*). In other words, it tested whether the indirect effect of depression (*X*) on QoL (*Y*) mediated by caring burden (*M*) was moderated by the relationship with the patient (*W*). To further understand the moderating effect of the relationship with the patient, a simple slope analysis was conducted. In the moderated mediation analysis, the caregiver’s age and living with the patient were controlled. The indirect effects were examined using 95% confidence intervals (CI) with 5000 bootstrap samples. If zero was not included within the 95% CI, the indirect effects were considered significant [23].

## 3. Results

### 3.1. Descriptive Statistics

The mean age of the caregivers was 52.1 (SD: 15.4) years; they were mostly female (72.0%), 40.9% were the patient’s spouse, and 63.4% lived with the patient. Furthermore, 47.3% of the caregivers were currently employed, and 53.8% were caring for inpatients. The caregivers in this study reported relatively high levels of depression (mean = 17.6), and more than half of them (51.6%) reported clinically significant depression (Table 1).

QoL according to caregivers’ characteristics is shown in Table 1. Patients’ spouses had a lower QoL than other family members (t = −4.28, *p* < 0.001), and caregivers who lived with the patient had a lower QoL than those who did not (*t* = −2.98, *p* = 0.004).

### 3.2. Results of the Correlation Analysis

The bivariate correlation coefficients are presented in Table 2. Caregivers’ QoL was positively related to being the patient’s spouse (r = 0.42, *p* < 0.001) and living with the patient (r = 0.29, *p* = 0.005), and negatively related to the caregivers’ depression (r = −0.85, *p* < 0.001) and caring burden (r = −0.75, *p* < 0.001). Caring burden was positively correlated with the caregivers’ age (r = 0.24, *p* = 0.019) and depression (r = 0.62, *p* < 0.001), and negatively correlated with being the patient’s spouse (r = −0.40, *p* < 0.001) and living with the patient (r = −0.27, *p* = 0.005).

### 3.3. Moderated Mediation Models

#### 3.3.1. Mediation Model for Quality of Life

**Figure 2:**
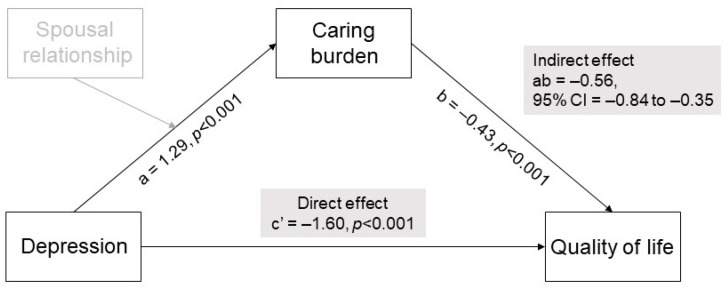
The mediating effect of caring burden on the relationship between depression and quality of life (Model 4).

Figure 2 shows the results of the mediation models. To examine the direct and indirect effects of caregivers’ depression on QoL through caring burden, the results of each path coefficient were generated by a series of multiple regressions conducted by Model 4 in PROCESS. After controlling for caregivers’ age and living with the patient, (a) the direct effect of depression on caring burden was significant (B = 1.29, *p* < 0.001); (b) the direct effect of caring burden on QoL was significant (B = −0.43, *p* < 0.001) and (c) the direct effect of depression on QoL was significant (B = −1.60, *p* < 0.001). In addition, the indirect effect of caregivers’ depression on QoL through caring burden was significant (ab = −0.56, 95% CI −0.84 to −0.35). These results indicate that the caring burden significantly mediates the association between depression and QoL in caregivers. Furthermore, the mediating effect accounted for 72.6% of the total effect (*p* < 0.001).

#### 3.3.2. Moderated Mediation Model for Quality of Life

**Figure 3:**
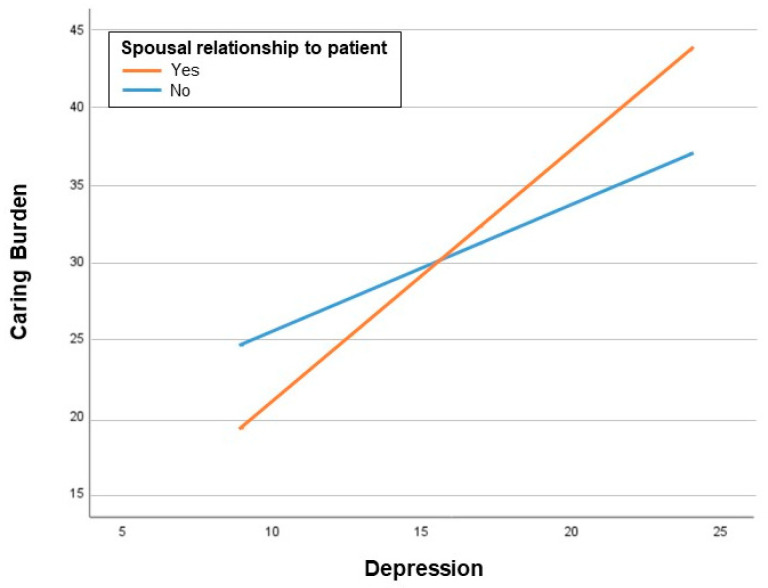
The moderating effect of spousal relationship with the patient on the correlation between depression and caring burden.

Conditional process analysis using Model 7 in PROCESS was used to test the moderated mediation assumptions. First, as shown in Table 3, the interaction between depression and the relationship with the patient exerted a significant effect on the caring burden (B = −0.80, *p* = 0.042), which means that the effect of depression on the caring burden was significantly different depending on the caregivers’ relationship with the patient. Specifically, the impact of depression on the caring burden for spouses of the patients was greater than that for caregivers who were not the patient’s spouse, as illustrated in Figure 3 (spousal relationship with patient: B = 1.63, *p* < 0.001; non-spousal relationship with the patient: B = 0.83, *p* = 0.005). The moderating effect of the relationship with the patient provided support for the theoretical argument that depression interacts with the caregiver’s relationship with the patient to influence the caring burden, which in turn impacts QoL.

The indirect effect of depression on QoL was significant for all relationships between patients and caregivers: (a) spousal relationship with the patient (B = −0.70, 95% CI −1.03 to −0.44); (b) non-spousal relationship with the patient (B = −0.36, 95% CI −0.67 to −0.12). Moreover, a test of equality of the conditional indirect effect of moderation, the Index of Moderated Mediation [28], was significant, indicating significant differences between spousal and non-spousal relationships with the patient in the depression *→* caring burden *→* QoL model (B = 0.35, 95% CI 0.04 to 0.66).

## 4. Discussion

This study explored the effect of depression on QoL in family caregivers of cancer patients. First, as expected, the effect of depression on caregivers’ QoL was mediated by the caregiving burden. This finding is supported by previous studies that showed that higher depression among family caregivers is associated with increased caregiving burden [4,12] and poor QoL [4,7,10].

In the current study, family caregivers had a clinical depression rate of 51.6%. Family caregivers of patients with cancer cannot access information and resources due to lack of time and rest, and a lack of professional knowledge and skills for cancer management due to various caring tasks, which may affect depressive morbidity [6]. Higher levels of depression interfere with the ability to provide care [16], affecting not only their role as caregivers but also the caregivers’ QoL [10]. By identifying family caregivers who are vulnerable to depression, professional psychological support can be provided to encourage informal caregivers to express their feelings and relieve the burden of care. In addition, if healthcare providers understand the aspects of the therapeutic roles of family caregivers for cancer patients, they can provide appropriate information to enable caregivers to perform well, which will help improve caregivers’ QoL.

According to previous studies, caregivers face increased difficulties in performing and managing their role as caregivers in addition to their existing roles related to parenting and employment; moreover, the caring burden may increase due to a lack of social support [29]. Although most caring experiences have a negative effect, it is suggested that the relationship between caring burden and QoL can be mediated by positive coping strategies, such as improving relationships with patients and new perceptions of the value of life of caregivers [6]. Considering coping strategies that can reduce the burden of care, interventions, such as e-Health-based counseling and treatment guides, can improve access to treatment and enhance the positive aspects of the care experience, thereby reducing the caring burden and improving QoL [30]. It is also suggested that available resources, such as home care and psychosocial support, can help improve the QoL of family caregivers.

Second, the spousal relationship with the patient moderated the indirect effect of depression on caregivers’ QoL. In the case of caregivers who were the patient’s spouse, the higher the depression, the higher the caring burden, which was associated with lower QoL. Caregiving spouses appear to have a greater risk of depression than other family caregivers [7,20]. As spouses provide full-time care and are more likely to be closer to the patient than other family members, the spouses’ caring experience may be different from that of the patient’s adult children, parents, and sibling [31]. When planning family caregiver management, the patient’s spouse should be subject of early identification. Berg and Upchurch [32] suggested that for shared stress events such as cancer, couples influence each other and coexist and adapt to dyads. In planning family caregiver support, the patient’s spouse should consider the dyadic approach of symptom management, skill acquisition, and psychological support interventions to improve QoL shared by patients and spouses, rather than individual caregivers’ QoL.

Overall, the results of this study suggest that identifying the mechanisms affecting the QoL among caregivers of cancer patients can help improve caregiver QoL, and highlight the need for interventions to manage caregiver depression and caring burden. Managing caregivers’ depression can have a positive effect on their health and ability to provide care. Further research is needed on the development of customized intervention programs according to their relationship with the patient to identify depression among caregivers, reduce the caring burden, and improve QoL.

This study has several limitations. First, clinical characteristics, including the patient’s cancer type, cancer stage, information on current treatment, or cancer patients’ performance, were not included in this study. All of these factors can potentially affect the caregiver’s QoL [10]. For example, the higher the Eastern Cooperative Oncology Group (ECOG) Performance Status [33] of cancer patients, the lower their QoL. In addition, pain and anxiety in patients were not included as measurable variables. Pain is one of the most common symptoms in cancer patients [34], and anxiety can occur in both patients and their spouses, which is known to have an immense impact on patients and their spouses’ QoL [35,36]. Further research is needed to better understand the impact of these variables on the QoL of family caregivers of cancer patients. Finally, the CQOLC-K is a multidimensional evaluation tool for caregivers’ QoL. QoL domain analysis can provide valuable information regarding specific aspects of health and well-being. Thus, we propose a follow-up study to develop practical interventions to improve caregivers’ QoL.

Despite these limitations, this study identified the factors and mechanisms affecting QoL in family caregivers of cancer patients by expanding the mediation model, including moderated effects, and providing important implications for future studies and interventions.

## 5. Conclusions

Depression among caregivers of cancer patients is on the rise, which leads to reduced QoL among caregivers. The results of this study suggest that caregivers’ depression is a stressor that triggers role-strain responses such as caring burdens. Our study supports the direct and indirect effects (via caring burden) of depression on QoL. Furthermore, caregivers’ relationship with patients moderated the association between caring burden and QoL. Caregivers who are the patients’ spouses may be at the greatest risk of depression. Therefore, it is necessary to identify caregivers’ level of depression and their relationship with the patient and implement educational programs and interventions for practically usable coping skills that can reduce the burden of care. We also propose that a multidisciplinary research strategy should be established to improve the QoL of caregivers of patients with cancer.

## Figures and Tables

**Table 1:** Quality of life according to caregivers’ general characteristics.

Variables	n (%)	M ± SD	*t*	*p*
Age		52.1 ± 15.4		
Gender			0.26	0.797
Male	26 (28.0)	76.04 ± 16.70		
Female	67 (72.0)	74.82 ± 21.72		
Spouse of the patient			−4.28	<0.001
Yes	38 (40.9)	64.95 ± 20.34		
No	55 (59.1)	82.22 ± 17.28		
Living with the patient			−2.98	0.004
Yes	59 (63.4)	70.73 ± 20.52		
No	34 (36.6)	82.85 ± 17.89		
Education			−1.87	0.065
~High school	51 (54.8)	71.63 ± 23.13		
Above college	42 (45.2)	79.45 ± 15.60		
Employment status			−0.07	0.943
Employed	44 (47.3)	75.00 ± 20.25		
Not employed	49 (52.7)	75.31 ± 20.67		
Care setting of patient			0.88	0.382
Outpatient	43 (46.2)	77.16 ± 20.00		
Inpatient	50 (53.8)	73.44 ± 20.71		
CES-D score		17.6 ± 7.8	9.72	<0.001
>16	45 (48.4)	90.09 ± 14.58		
≤16	48 (51.6)	61.17 ± 14.11		
Caring burden		33.4 ± 17.3		
Quality of life		75.2 ± 20.4		

**Table 2:** Bivariate correlations between study variables.

Variables	1	2	3	4	5	6	7	8	9
	r (*p*)	r (*p*)	r (*p*)	r (*p*)	r (*p*)	r (*p*)	r (*p*)	r (*p*)	r (*p*)
1. Age	1								
2. Gender *	0.13 (0.211)	1							
3. Spouse of a patient *	−0.60 (<0.001)	−0.03 (0.772)	1						
4. Living with a patient *	−0.25 (0.017)	−0.03 (0.815)	0.54 (<0.001)	1					
5. Education *	−0.31 (0.003)	−0.14 (0.564)	0.36 (<0.001)	0.54 (<0.001)	1				
6. Employment status *	0.28 (0.006)	0.42 (<0.001)	−0.17 (0.095)	0.36 (<0.001)	0.04 (0.720)	1			
7. Care setting of patient *	−0.01 (0.989)	0.14 (0.171)	0.02 (0.858)	−0.17 (0.095)	−0.01 (0.514)	0.03 (0.787)	1		
8. Depression	0.10 (0.324)	0.04 (0.700)	0.04 (0.700)	0.02 (0.858)	−0.31 (0.003)	−0.01 (0.927)	0.09 (0.396)	1	
9. Caring burden	0.24 (0.019)	−0.04 (0.735)	−0.04 (0.735)	−0.41 (<0.001)	−0.11 (0.185)	−0.07 (0.523)	−0.00 (0.981)	0.62 (<0.001)	1
10. Quality of life	−0.16 (0.134)	−0.27 (0.797)	0.42 (<0.001)	0.29 (0.005)	0.19 (0.065)	0.01 (0.943)	−0.09 (0.382)	−0.85 (<0.001)	−0.75 (<0.001)

Note: * Correlations between binary and quantitative variables were calculated using point-biserial correlations. Gender: (Male = 0, Female = 1), Spouse of a patient (Yes = 0, No = 1), Living with the patient (Yes = 0, No = 1), Education (High school = 0, More than college = 1), Employment status (Employed = 0, Not employed = 1), care setting of patient (outpatient = 0, inpatient = 1).

**Table 3:** Indirect effect of depression on quality of life mediated by caring burden and moderated by spousal relationship with patient (Model 7).

Predictors	Caring Burden (*M*)			Quality of Life (*Y*)		
	B (SE)	95% CI	*p*	B (SE)	95% CI	*p*
Depression (*X*)	1.63 (0.27)	1.10 to 2.16	<0.001	−1.60 (0.16)	−1.91 to −1.28	<0.001
Caring burden (*M*)				−0.43 (0.07)	−0.58 to −0.29	<0.001
Spousal relationship (*W*)	12.59 (8.31)	−3.93 to 29.12	0.133			
Depression × Spousal relationship (*X* × *W*)	−0.80 (0.39)	−1.58 to −0.03	0.042			
R^2^	0.45			0.80		
F	14.20			90.20		
*p*	<0.001			<0.001		
**Difference between conditional indirect effect**	**Index**	**B (SE)**	**95% CI**			
Spousal relationship (*X→M→Y*)	0.35	0.16	0.04 to 0.66			

Note: 95% CI, 95% bootstrapped confidence intervals; *M*, mediator; SE, standard error; *W*, moderator; *X*, independent variable; *Y*, outcome variable.

## Data Availability

The datasets used or analyzed during the current study are available from the corresponding author upon reasonable request.
